# Identification of Dysregulated Expression of G Protein Coupled Receptors in Endocrine Tumors by Bioinformatics Analysis: Potential Drug Targets?

**DOI:** 10.3390/cells11040703

**Published:** 2022-02-17

**Authors:** Valentine Suteau, Mathilde Munier, Rym Ben Boubaker, Méline Wery, Daniel Henrion, Patrice Rodien, Claire Briet

**Affiliations:** 1 Département d’Endocrinologie-Diabétologie Nutrition, Centre Hospitalier Universitaire (CHU) d’Angers, 49933 Angers, France; mathilde.munier@univ-angers.fr (M.M.); parodien@chu-angers.fr (P.R.); claire.briet@chu-angers.fr (C.B.); 2 Laboratoire MITOVASC, UMR CNRS 6015, INSERM 1083, Université d’Angers, 49100 Angers, France; rym.benboubaker@univ-angers.fr (R.B.B.); daniel.henrion@univ-angers.fr (D.H.); 3 Centre de Référence des Maladies Rares de la Thyroïde et des Récepteurs Hormonaux, Centre Hospitalier Universitaire (CHU) d’Angers, 49933 Angers, France; 4 Faculté de Santé, Université d’Angers, SFR ICAT, 49100 Angers, France; meline.wery@univ-angers.fr; 5 Centre de Référence des Maladies Rares de l’Hypophyse, Centre Hospitalier Universitaire (CHU) d’Angers, 49933 Angers, France

**Keywords:** G protein-coupled receptors, endocrine tumors, paraganglioma, pheochromocytoma, adrenocortical cancer, medullary thyroid cancer, pituitary adenoma, drug repurposing

## Abstract

Background: Many studies link G protein-coupled receptors (GPCRs) to cancer. Some endocrine tumors are unresponsive to standard treatment and/or require long-term and poorly tolerated treatment. This study explored, by bioinformatics analysis, the tumoral profiling of the GPCR transcriptome to identify potential targets in these tumors aiming at drug repurposing. Methods: We explored the GPCR differentially expressed genes (DEGs) from public datasets (Gene Expression Omnibus (GEO) database and The Cancer Genome Atlas (TCGA)). The GEO datasets were available for two medullary thyroid cancers (MTCs), eighty-seven pheochromocytomas (PHEOs), sixty-one paragangliomas (PGLs), forty-seven pituitary adenomas and one-hundred-fifty adrenocortical cancers (ACCs). The TCGA dataset covered 92 ACCs. We identified GPCRs targeted by approved drugs from pharmacological databases (ChEMBL and DrugBank). Results: The profiling of dysregulated GPCRs was tumor specific. In MTC, we found 14 GPCR DEGs, including an upregulation of the dopamine receptor (*DRD2*) and adenosine receptor (*ADORA2B*), which were the target of many drugs. In PGL, seven GPCR genes were downregulated, including vasopressin receptor (*AVPR1A*) and PTH receptor (*PTH1R*), which were targeted by approved drugs. In ACC, *PTH1R* was also downregulated in both the GEO and TCGA datasets and was the target of osteoporosis drugs. Conclusions: We highlight specific GPCR signatures across the major endocrine tumors. These data could help to identify new opportunities for drug repurposing.

## 1. Introduction

G protein-coupled receptors (GPCRs) are the largest family of membrane receptors involved in many types of cellular responses. The GPCR family represents approximately 4% of the human genes, with more than 800 members [1]. GPCRs are involved in important functions, such as cardiac function, hormone regulation, immune responses and neurotransmission. Abnormal expression or activity is associated with several human diseases [2]. As a result, GPCRs are considered as therapeutic targets in many diseases. Drugs that target GPCRs account for about 34% of the current medicines, making it the largest family of validated pharmacological targets [3], with cumulative sales for 2011 to 2015 of $890 billion in the United States [4]. The pharmacological targeting of GPCRs is a well-established approach for treatment in many human diseases.

Recent evidence supports the involvement of many GPCRs and their ligands in controlling the initiation and progression of tumors (cell proliferation, metastasis, adhesion or angiogenesis) [5,6,7]. Recently, many studies revealed that GPCRs are mutated and/or their expression dysregulated in multiple cancers [5,8]. Therefore, GPCRs can be considered as attractive targets for novel therapeutic treatments of tumors or for the repurposing of approved drugs with a target-based approach.

Bioinformatics approaches have allowed for the identification of potential therapeutic and/or prognostic targets in cancer. The Gene Expression Omnibus (GEO) and The Cancer Genome Atlas (TCGA) databases are the most frequently used to identify differentially expressed genes (DEGs). GEO is a database repository of microarrays gene expression data. The Cancer Genome Atlas (TCGA) is a project that has generated publicly available genomic and clinical data for various types of cancer [9]. GPCRomic studies have been conducted for the most frequent cancers, such as prostate or breast cancer [7,10,11]. In addition, the team of P. Insel focused more specifically on pancreatic adenocarcinoma [10,12,13]. Moreover, we recently established an atlas of GPCRs in radioactive iodine-resistant thyroid cancer by compiling data from the GEO database, TCGA cohort and our transcriptomic analysis [14]. However, the dysregulation of GPCR expression has not been investigated in depth in other endocrine tumors.

Endocrine tumors include tumors in glands that produce hormones (thyroid, parathyroid, pituitary, adrenal gland) and those originated from neuroendocrine cells. Medullary thyroid cancer (MTC) is a neuroendocrine tumor and arises from parafollicular cells or C cells. For patients with progressive and multi-metastatic MTC, the first-line systemic treatment is represented by multiple tyrosine kinase inhibitors (cabozantinib, vandetanib). These treatments have shown transient and/or partial efficacy with numerous side effects. However, there is a lack of evidence for the use of other therapies, such as chemotherapy or metabolic radiotherapy. Pituitary neuroendocrine tumors (PitNETs), traditionally designated as pituitary adenomas, include different groups of neoplasms of anterior pituitary cell origin [15]. They are classified according to the expression pattern of anterior pituitary hormones, the hormone hypersecretion and the tumor mass [16]. PitNETs are mostly well controlled after surgery. However, these tumors may exhibit aggressive behavior, with resistance to conventional treatments. The current chemotherapy, temozolomide, is effective in only one-third of the patients [17]. The same is true for primary malignant adrenal tumors, including pheochromocytoma (PHEO) and adrenocortical cancer (ACC). Pheochromocytomas (PHEOs) are tumors derived from the neural crest in the adrenal medulla. They are grouped with paragangliomas (PGLs), which share the same histological origin but are located in the paraganglia of the autonomic nervous system. The management of metastatic PHEO and PGL remains a major challenge since, to date, even though they represent less than 25% of cases, there are no curative treatment options [18]. For advanced ACC, besides surgical resection and loco-regional treatments, treatment with mitotane combined with chemotherapy (etoposide, doxorubicin and cisplatin) is often proposed according to prognostic parameters [19]. This treatment is both limited by systemic toxicity and by transient or partial efficacy.

These neuroendocrine tumors (NETs) share some features, such as slow growth and poor response to standard chemotherapies. To date, the somatostatine/somatostatine receptor (SSTR) is the only hormone-GPCR with an approved indication in the treatment of NETs. The identification of new therapeutic targets is required. We, therefore, used a GPCRomic approach to identify differentially expressed GPCRs genes in several endocrine tumors in order to identify GPCRs that may be new therapeutic targets for drug repurposing.

## 2. Materials and Methods

### 2.1. Collection of GEO Datasets and TCGA Data

To comprehensively evaluate GPCRs expression between endocrine tumors and non-tumoral tissues, we incorporated the expression data of GPCRs from GEO. We selected four types of endocrine tumors: medullary thyroid cancer (MTC), pituitary neuroendocrine tumors (PitNETs), pheochromocytoma (PHEO) and paraganglioma (PGL) and adrenocortical carcinoma (ACC). An electronic search of the GEO databases was carried out with the following key terms: “medullary thyroid”, “adrenocortical”, “pituitary” “pheochromocytoma”, “paraganglioma”, AND “cancer OR carcinoma OR tumor”. The GEO datasets published before 1 April 2020 were included. We filtered data by ‘Expression profiling by array’ in ‘*homo sapiens*’. The reference lists were manually reviewed for further identification of relevant studies. We selected the studies with the following inclusion criteria: studies contained mRNA expression data in tumoral and non-tumoral tissues in previous cited endocrine tumor. The exclusion criteria were: cell lines, xenograft studies, miRNA analyses, no comparison with normal tissue, duplicate. The microarray datasets were downloaded from the GEO database for each endocrine tumor and are summarized in Table 1. 

To validate transcriptomic analysis, we also obtained the GPCRs expression profile of human ACC from The Cancer Genome Atlas (TCGA, https://cancergenome.nih.gov/, accessed on 17 December 2021) and adrenal normal tissues from The Genotype-Tissue Expression Database (GTEX, https://gtexportal.org/home/, accessed on 17 December 2021) with online analysis tool (https://insellab.github.io/, accessed on 17 December 2021). Data for other endocrine tumors were not available.

### 2.2. Identification of Differentially Expressed Genes

DEGs between tumoral and normal samples were identified using the GEO2R web application (http://www-ncbi-nlm-nih-gov.proxy.insermbiblio.inist.fr/geo/geo2r/, accessed on 7 January 2022). GEO2R is an interactive online tool, which compares two groups of samples (i.e., normal vs. tumoral) to obtain genes with different expressions under the same experimental conditions. It uses Bioconductor R packages to transform and analyze GEO data with GEO query and limma analyses [36]. We applied adjustment to the P-values with the Benjamini & Hochberg method (false discovery rate, FDR) and a log_2_ transformation to the data. We saved the NCBI annotations where possible. Then, we filtered, from the set of genes studied, a list of 396 GPCRs (Table S1). The DEGs with an adjusted *p* < 0.05 were considered as the cut-off criteria. Then, to strengthen the significance of the data, we chose to select DEGs obtained from all independent datasets of tumor tissue samples rather than combine the GSE data, as in previous publications [11,37]. We applied for Venn software online (http://www.interactivenn.net/, accessed on 26 June 2020) to obtain the common DEGs in all independent cohorts for each endocrine tumor [38].

### 2.3. Identification of GPCRs as Targets for Approved Drugs

We evaluated drug targets among GPCRs differentially expressed between normal and tumoral samples and the list of approved drugs for each of these receptors. This was conducted using two pharmacological databases: ChEMBL and DrugBank. DrugBank is a clinically oriented drug database that provides information about drug and drug action for more than 500,000 drugs [39]. ChEMBL is an open database containing information extracted from the medicinal chemistry literature regarding the compounds tested and their primary target [40]. Approved drugs were verified with those listed by the Food and Drug Administration (FDA) (https://www.fda.gov, accessed on 3 January 2022) and by the European Medicines Agency (EMA) (https://www.ema.europa.eu, accessed on 3 January 2022).

### 2.4. Survival Analysis

The GEPIA database (http://gepia.cancer-pku.cn, accessed on 14 February 2022) was used for survival analysis of the dysregulated GPCR genes. Data from TCGA-ACC project (88 patients with ACC) were analyzed. To analyze the prognostic value of GPCR genes, the patient samples were split into 2 groups according to the median expression of the biomarker. The two patient cohorts were compared by a Kaplan–Meier survival plot, and the logrank P value was calculated.

## 3. Results

### 3.1. Medullary Thyroid Cancer

For medullary thyroid cancer (MTC), only one study was available from the GEO database (GSE27155). The samples consisted of four normal thyroid tissues and two medullary carcinomas. The analysis revealed 14 DEGs for GPCRs (seven downregulated and seven upregulated) (Table 2). The TSH receptor (*TSHR*) was the main receptor downregulated in MTC compared to normal thyroid tissue (log_2_FC: −2.22) as well as *GPRC5A*; an orphan receptor was also found to be downregulated (log_2_FC: −1.93). For the upregulated GPCRs, the majority were weakly upregulated, such as dopamine receptor (*DRD2,* log_2_FC: 0.62) and cholecystokinin receptor (*CCKBR*, log_2_FC: 0.59). The two most upregulated GCPRs compared to normal thyroid tissue were the adhesion receptor *ADGRG2* (log_2_FC: 1.42) and the receptor *LGR5* (Leucine Rich Repeat Containing G Protein-Coupled Receptor 5, log_2_FC: 1.43).

### 3.2. Pituitary Neuroendocrine Tumors (PitNETs)

**Figure 1:**
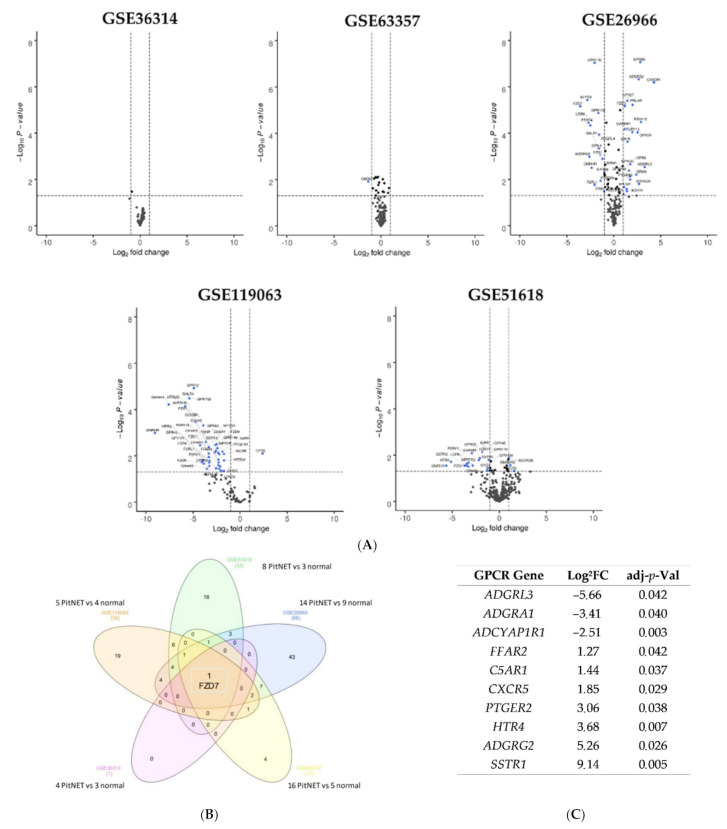
Dysregulated GPCR in pituitary neuroendocrine tumors. (**A**) Volcano plots for GSE119063, GSE51618, GSE26966, GSE63357 and GSE36314. Each GPCR transcript was represented by a spot. Log_2_ fold change was plotted against the −log_10_ *p*-value (<0.05, horizontal line). Differentially expressed GPCR genes were selected with adjusted *p* < 0.05 among the mRNA expression profiling datasets; (**B**) Venn diagram for GSE119063, GSE51618, GSE26966, GSE63357 and GSE36314 datasets. For each dataset, the number of significant DEGs was indicated in brackets. One GPCR gene (*FZD7*) was identified in the five datasets; (**C**) list of DEGs selected from analysis of invasive vs. non-invasive PitNETs in GSE51618 datasets. Abbreviation: PitNETs (pituitary neuroendocrine tumors), FC, fold-change.

For PitNETs, five gene expression profiles (GSE119063, GSE51618, GSE36314, GSE63357, GSE36966) were acquired from the GEO database. The dataset contained from four to sixteen tumor samples and from three to nine normal samples. The datasets consisted of several pathological subtypes of PitNETs (prolactinoma, non-functioning or GH adenoma). To be more representative, a specific pathological type was not selected. The analysis of these datasets revealed from one to sixty-six DEGs (Figure 1, Table S2). Among them, the Frizzled 7 receptor *FZD*7 was downregulated in the five datasets (GSE119063, log_2_FC = −4.26, *p* = 0.004, GSE51618, log_2_FC = −3.7, *p* = 0.029, GSE36314, log_2_FC = −0.88, *p* = 0.034, GSE63357, log_2_FC = −0.70, *p* = 0.009, GSE36966, log_2_FC = −3.57, *p* < 0.001). Interestingly, in the GSE51618 dataset, we could also compare the expression profile between invasive and non-invasive nonfunctional pituitary adenomas. *SSTR1* was highly upregulated in invasive adenoma (log_2_FC = 9.14, *p* = 0.005) as well as the adhesion receptor *ADGRG2* (log_2_FC = 5.26, *p* = 0.026), the serotonin receptor *HTR4* 5 (log_2_FC = 3.68, *p* = 0.007) and the prostaglandin receptor *PTGER2* (log_2_FC = 3.06, *p* = 0.038). Conversely, adhesion receptors *ADGRA1* and *ADGRL3* were downregulated in invasive tumors (log_2_FC = −3.41, *p* = 0.040, log_2_FC = −5.66, *p* = 0.042, respectively) (Figure 1).

### 3.3. Pheochromocytoma and Paraganglioma

Four gene expression profiles (GSE50442, GSE39716, GSE19422, GSE60459) were acquired from the GEO database for pheochromocytoma and paraganglioma. The dataset contained from two to sixty-one pheochromocytoma samples, four to twenty-four paraganglioma and from three to eight normal samples (medullar adrenal tissue). All PHEOs were non-metastatic. For PGL, GSE39716 and GSE60459 included ten and three metastatic paraganglioma, respectively.

**Figure 2:**
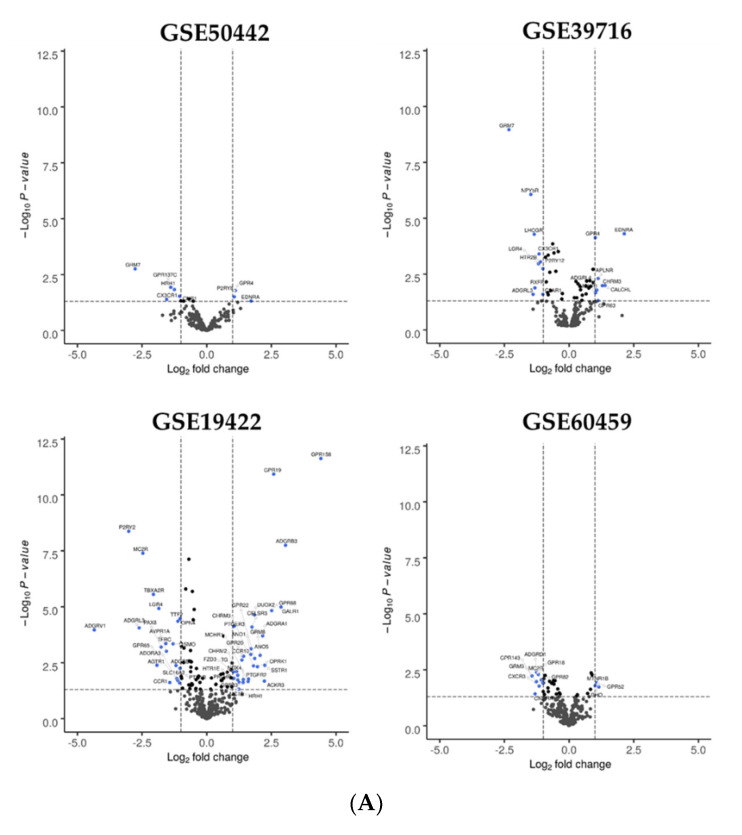

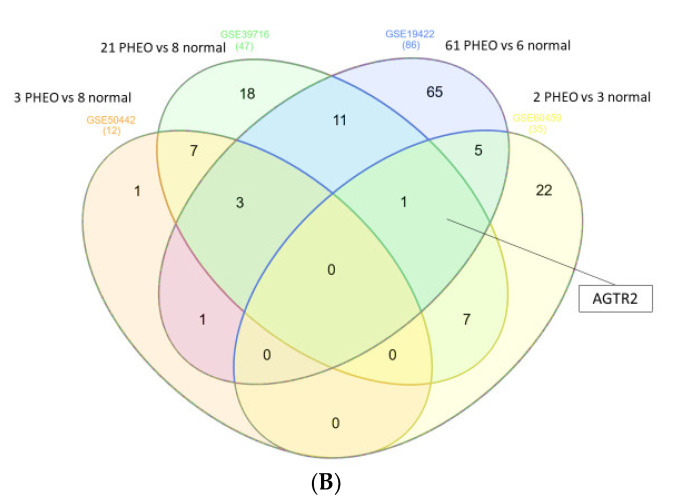
Dysregulated GPCR in pheochromocytoma. (**A**) Volcano plots for GSE50442, GSE39716, GSE19422, GSE60459. Each GPCR transcript was represented by a spot. Log_2_ fold change was plotted against the −log_10_ *p*-value (*p* < 0.05, horizontal line). Differentially expressed GPCR genes were selected with adjusted *p* < 0.05 among the mRNA expression profiling datasets; (**B**) Venn diagram for GSE50442, GSE39716, GSE19422, GSE60459 datasets. For each dataset, the number of significant DEGs was indicated in brackets. Abbreviation: FC, fold-change.

The data for PHEO were quite heterogeneous among the four studies. The number of GPCRs with a significant difference in expression between healthy and tumor tissue varied between 12 and 86 (Figure 2, Table S3). Although there was no receptor significantly common to all four series, the angiotensin II type 2 receptor (*AGTR2*) was found to be downregulated in the tumor tissue in all the studies (GSE60459, log_2_FC = −1.1, *p* = 0.03; GSE50442, log_2_FC = −0.56, *p* = 0.493; GSE39716, log_2_FC = −0.75, *p* = 0.003; GSE19422, log_2_FC = −0.60, *p* = 0.003).

**Figure 3:**
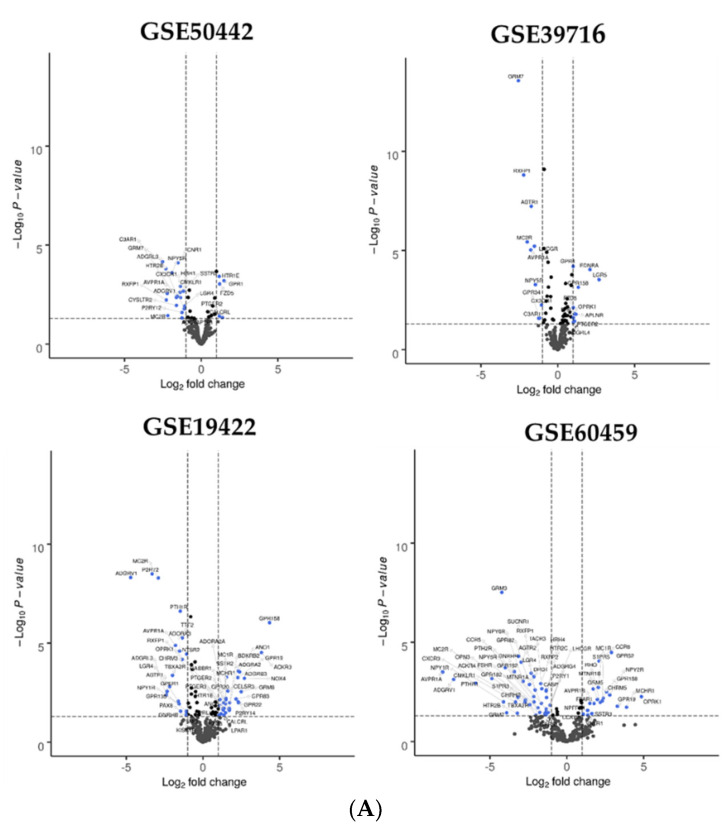

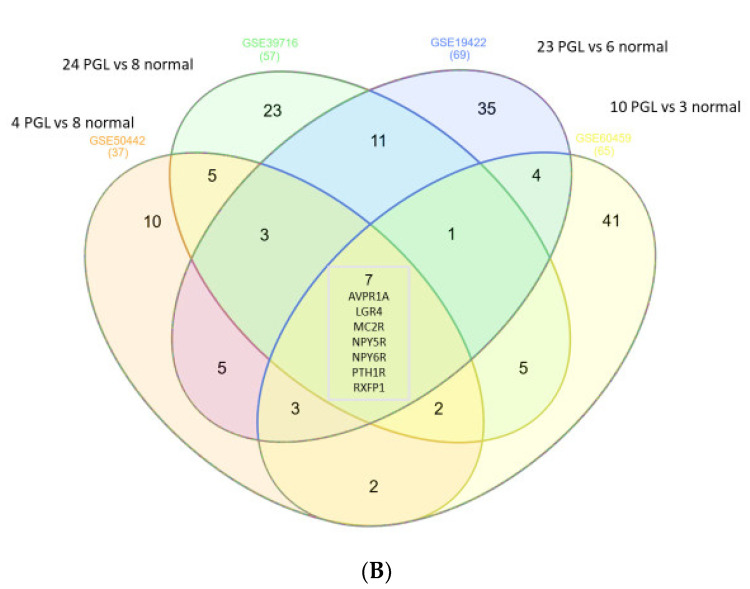
Dysregulated GPCR in paraganglioma. (**A**) Volcano plots for GSE50442, GSE39716, GSE19422, GSE60459. Each GPCR transcript was represented by a spot. Log_2_ fold change was plotted against the −log10 *p*-value (*p* < 0.05, horizontal line). Differentially expressed GPCR genes were selected with adjusted *p* < 0.05 among the mRNA expression profiling datasets; (**B**) Venn diagram for GSE50442, GSE39716, GSE19422, GSE60459 datasets. For each dataset, the number of significant DEGs was indicated in brackets. Seven GPCR genes were identified in the four datasets. Abbreviation: PGL: paraganglioma, FC, fold-change.

In PGL, the analysis revealed from 37 to 69 DEGs for GPCRs. Venn diagram analysis revealed that seven GPCRs were down regulated compared with normal adrenal gland in the four datasets (*AVPR1A*, *MC2R*, *NPY5R*, *NPY6R*, *RXFP1*, *LGR4*, *PTH1R*) (Figure 3, Table S4). When we compared metastatic PGL and non-metastatic PGL in GSE39716 and GSE60459, no significant DEG were found.

### 3.4. Adrenocortical Carcinomas

**Figure 4:**
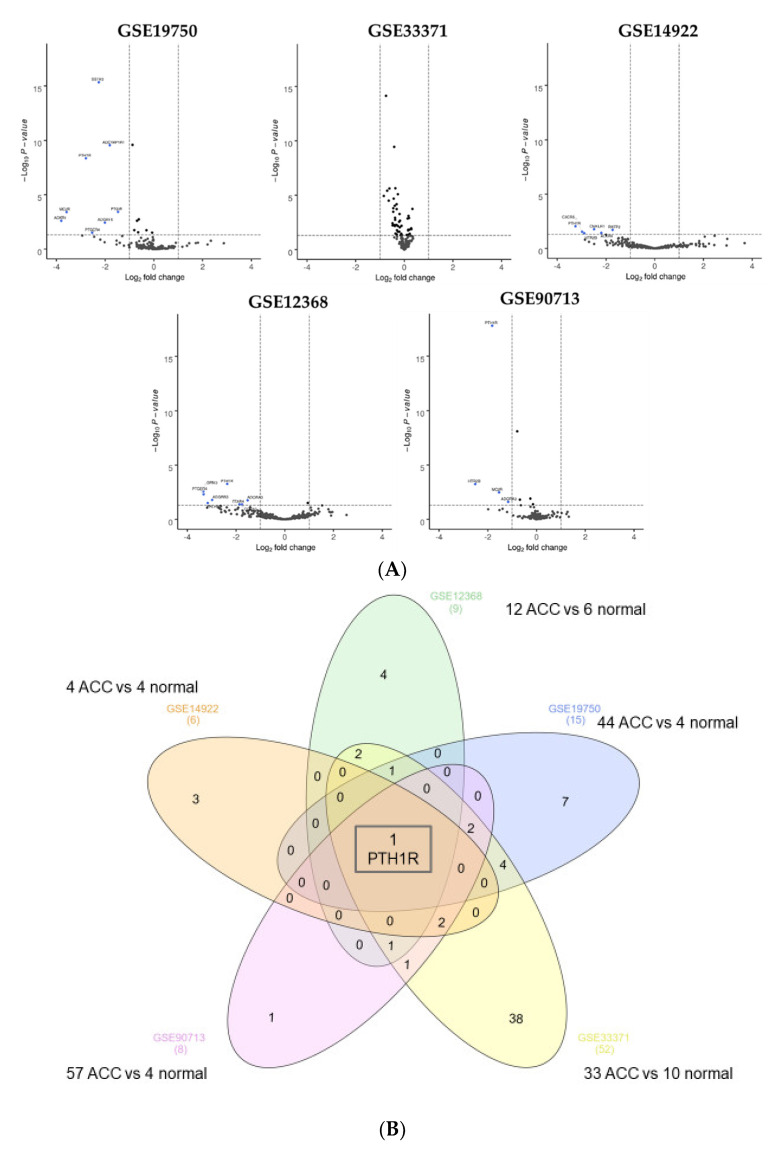
Dysregulated GPCR in adrenocortical cancer. (**A**) Volcano plots for GSE14922, GSE12368, GSE19750, GSE33371, GSE90713. Each GPCR transcript was represented by a spotLog_2_ fold change plotted against the −log10 *p*-value (*p* < 0.05, horizontal line). Differentially expressed GPCR genes were selected with adjusted *p* < 0.05. Differentially expressed GPCR genes were selected with adjusted *p* < 0.05 among the mRNA expression profiling datasets; (**B**) Venn diagram for GSE14922, GSE12368, GSE19750, GSE33371, GSE90713 datasets. For each dataset, the number of significant DEGs was indicated in brackets. One GPCR gene (*PTH1R*) was identified in the five datasets. Abbreviation: FC, fold-change.

For ACC, five gene expression profiles (GSE14922, GSE90713, GSE12368, GSE19750, GSE33371) were acquired from the GEO database. The dataset contained from four to fifty-seven cancer samples and from four to ten normal samples. The analysis of these datasets revealed six, eight, fifteen, nine and fifty-two DEGs, respectively. Venn diagram analysis revealed that *PTH1R* was the only gene downregulated in the five datasets (Figure 4, Table S5). To validate this finding, TCGA data (TCGA-ACC Project) were used to quantify differential expression of *PTH1R* by comparing tumors against adrenal normal tissue from the Gene Tissue Expression Project (GTEx) database. In the TCGA cohort, *PTH1R* was also downregulated (log_2_FC: −3.37, *p* < 0.0001).

### 3.5. Identification of Dysregulated G Protein Coupled Receptors as Targets for Approved Drugs

Among the dysregulated GPCR, we determined which receptors were targeted by approved drugs and how many drugs were available for these targets. The data were summarized in the Table 3. In MTC, among the 14 DEG, *CCKBR*, *D2R* and Adenosine A2B receptor (*ADORA2B*) were the target of drugs. *DRD2* was the most frequently targeted receptor with over 40 anti-psychotic agents acting as *DRD2* antagonists. For PitNET, any drug did not target *FZD7*. Interestingly, three up-regulated GPCR in invasive PitNET were targeted receptor: *SSTR1*, *HTR4* and *PTGER2*. Among the seven down-regulated GPCRs in paraganglioma, Arginine Vasopressin Receptor 1A (*AVPR1A*), ACTH receptor, *MC2R,* and *PTH1R* were the target of 2 to 4 drugs each. *PTH1R* also found to be down-regulated in adrenocortical cancer, was targeted by 3 agonists used in osteoporosis or hypoparathyroidism.

### 3.6. Survival Analysis

**Figure 5:**
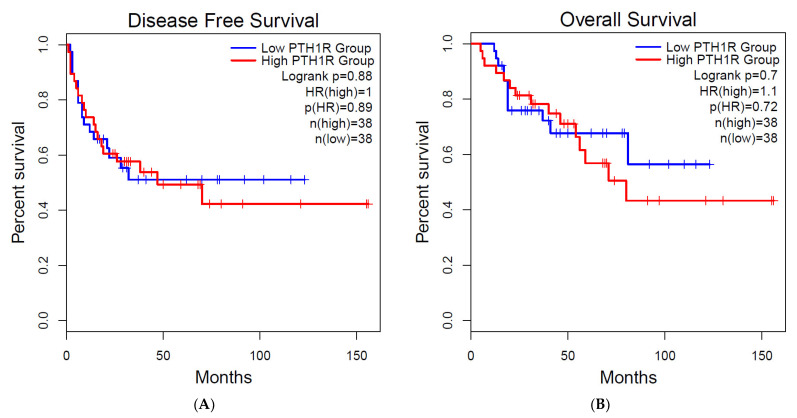
Survival analysis for *PTH1R* in adrenocortical cancer. To analyze the prognostic value of *PTH1R* gene expression, the patient samples are split into 2 groups according to the median expressions of the genes. The 2 patient cohorts were compared by a Kaplan–Meier survival plot, and log-rank *p* value was calculated. (**A**) Disease free survival; (**B**) overall survival.

Survival data were only available for ACC from TCGA. We, therefore, chose to study the gene expression of *PTH1R* for ACC. Using the GEPIA database to explore the association between gene expression and survival, we found that *PTH1R* expression was neither significantly correlated with overall survival nor disease-free survival of ACC patients (log-rank *p* = 0.72 and log-rank *p* = 0.88, respectively) (Figure 5).

## 4. Discussion

This study constitutes the first atlas of GPCRs within major endocrine tumors after thyroid cancer [14]. Drug repurposing strategies provided a new approach for revealing new clinical applications of an approved drug and for developing antitumor drugs. The use of GPCRomic approaches leads to the discovery of GPCRs that could contribute to cancer pathophysiology and, thus, may be therapeutic targets.

### 4.1. Medullary Thyroid Cancer

We confirmed the upregulation of cholecystokinin 2 receptor (*CCKBR*/*CCK2R*) expression in MTC, which is targeted by radiolabeled peptide analogs for molecular imaging and targeted radiotherapy of different human tumors, such as MTC [41,42]. The dopamine receptor *DRD2* was also upregulated. It is known that L-dopa inhibits calcitonin secretion in MTC, and a new chimeric somatostatin-dopamine analog has recently shown an anti-tumor effect in vitro [43]. Interestingly, *DRD2* is the target of many drugs already approved and is, therefore, accessible for drug repurposing. Like *DRD2*, the adenosine receptor *ADORA2B* was upregulated and the target of approved drugs. However, sparse data are available on the role of these treatments in thyroid cancer, although previous studies have shown a role for adenosine receptors in calcitonin secretion in C-cells [44]. *ADGRG2*/*GPR64* was also over-expressed in MTC (log_2_FC = 1.42, *p* = 0.0049) as in a number of carcinomas derived from breast cancer, parathyroid tumors, Ewing sarcomas, prostate, kidney or lung, and the inhibition of its expression promotes invasiveness and metastatic spread [45,46]. *GPRC5A* was downregulated (log_2_FC = 1.93, *p* = 0.0002) as previously reported in other cancers, especially in lung cancer, where it displayed a tumor suppressive role [47]. *FZD1* was also downregulated as previously described in non-medullary thyroid cancer, in which inhibition increased invasiveness, indicating a possible pathogenesis role [48]. However, to have only one study with a very small number of samples limits the significance of the findings for medullary thyroid cancer. In addition, the different histological origin between the normal tissue (mainly of follicular origin) and tumor tissue (medullary origin) probably explains part of the results. This is the case, for example, with the downregulation of the TSH receptor (*TSH-R*), which is strongly expressed in normal thyroid tissue but was negligible in MTC, as expected [49].

### 4.2. Pituitary Neuroendocrine Tumors (PitNETs)

Five studies were available in the GEO database comparing pituitary adenomas and the normal pituitary. Data regarding aggressiveness were not available. Independent of tumor characteristics (secreting or non-secreting, invasive or non-invasive), Frizzled receptor 7 (*FZD7*) was downregulated in PitNETs. Interestingly, in invasive adenomas compared to the normal pituitary gland (GSE51618) *FZD7* was also found to be downregulated. To date, this data was not described before in the literature. However, *SFRP2* and *4* (secreted-frizzled related protein), Wnt antagonists, considered as tumor suppressor genes, were downregulated in pituitary adenomas, in particular in invasive tumors, suggesting a role of the Wnt pathway in the progression of PitNETs [50,51,52]. Among the differentially expressed GPCR genes between non-invasive and invasive tumors, the somatostatin type 1 receptor (*SSTR1*) was known and already targeted by somatostatin analogs. *HTR4* and *PTGER2* were also targeted by agonists. The antitumoral and antiangiogenic effects of *HTR4* agonists were thus shown [53,54].

### 4.3. Pheochromocytoma or Paraganglioma

For PHEO, no receptor was significantly common to all four series. However, even if, in one study, angiotensin II type 2 receptor (*AGTR2*) was not statistically downregulated, it was found to be downregulated in the tumoral tissue in all the studies. Indeed, the absence or a very low expression of AGTR2 has been described in PHEO [55]. Interestingly, this receptor has been also described for its antiproliferative role in cancer, including in the pheochromocytoma PC12 cell line in response to angiotensin II [56]. However, unlike the angiotensin II type 1 receptor, no approved drug targets the *AGTR2*. In addition, it would be interesting to have metastatic pheochromocytomas to compare the profile between metastatic and non-metastatic, as for paragangliomas. However, no data were available.

For PGL, seven GPCRs were found to be downregulated when compared to normal medulla adrenal tissue. However, no data are available in the literature regarding their expression level or their role in paragangliomas. Interestingly, the arginine vasopressin receptor, *AVPR1A*, was also found to be downregulated in thyroid cancer and associated with progression-free survival, while it was upregulated in castration-resistant prostate cancer with an antitumoral effect of a selective AVPR1A antagonist [14,57]. Moreover, AVPR1A agonist and antagonists are approved drugs and could be tested as proof of concept. When we compared malignant paraganglioma and benign paraganglioma, no significant DEGs were found. The patient numbers were small in the two datasets, probably limiting the statistical power of the analyses.

### 4.4. Adrenocortical Carcinoma

In ACC, *PTH1R* was found downregulated in 5/5 datasets. This result was confirmed in ACC samples from the TCGA cohort. *PTH1R* is a major endocrine regulator of skeletal development and mineral homeostasis. It was also found that *PTH1R* enhanced the secretion of steroid hormones by human adrenocortical cells via a signaling mechanism involving the activation of both the Gs and Gq pathways [58]. *PTH1R* was previously immunolocalized in the cytoplasm in a normal adrenal cortex [59]. The *PTH1R* mRNA expression level was significantly higher in aldosterone-producing adenomas than in cortisol-producing adenoma [59] but seemed to be similar between ACCs and adenomas [60]. To the best of our knowledge, this is the first comparison between ACCs and a normal adrenal cortex. We found a downregulation of *PTH1R,* while PTHrp seemed to have a positive effect on the proliferation of the H295R lineage [60]. This could be explained by a downregulation of *PTH1R* by PTHrP, as described in vascular smooth muscle, osteoblastic or kidney cell lines [61]. The role of *PTH1R* in tumor progression is still poorly understood. Indeed, *PTH1R* knockdown increased cell migration and invasion and decreased tumor differentiation in neuroblastoma but had an opposite effect in osteoblastoma [62]. Finally, the *PTH1R* expression was neither significantly correlated with overall survival nor disease-free survival in ACC patients. However, there were only 88 patients in the study, probably limiting the statistical power of the analyses. Moreover, the absence of an association with survival should not, in our opinion, compromise potential therapeutic targets, as in thyroid cancer, where TSH inhibition influences survival even though its receptor expression level in the tumor is not associated with overall survival [14,63].

RNA-seq and microarrays that assess GPCR expression have certain limitations. First, RNA expression does not necessarily reflect protein expression. However, methods to assess GPCR protein expression are limited. Immunological techniques are limited by the lack of well-validated and specific GPCR antibodies [64]. The low expression of GPCRs compared to other proteins makes detection of GPCRs complicated. Moreover, recent studies found a closer relationship between the mRNA and protein, especially for GPCRs [65,66]. Another alternative to validate GPCR expression would be cell signaling and functional assays. Second, the clinical data were not associated with the microarrays data in the GEO repository, which limits the evaluation of the prognostic impact of these GPCRs on recurrence or progression. The data from the GPCRomic analyses need to be validated by further prospective studies for specific GPCRs with clinical evaluation.

## 5. Conclusions

This study presented all the data available to date regarding GPCRs in endocrine tumors. The data could help to identify new potential pathways worth targeting, eventually with approved drugs after proving the tumoral impact in preclinical models. The high-throughput screening of clinically relevant GPCRs with approved drugs would then be an interesting tool for the evaluation of these molecules in endocrine tumors.

## Figures and Tables

**Table 1:** Gene Expression Datasets from GEO Database.

Tumor Type	Series	Contributors	Samples	Platforms
Medullary Thyroid Cancer	GSE27155	Giordano TJ [20,21]	2 MTC vs. 4 normal	GPL96: Affymetrix Human Genome U133A Array
Pituitary Neuroendocrine Tumor	GSE36314	Oyesiku NM [22]	4 PitNET vs. 3 normal	GPL8300: Affymetrix Human Genome U95 Version 2 Array
	GSE119063	Wu Z (unpublished)	5 PitNET vs. 4 normal	GPL13607: Agilent-028004 SurePrint G3 Human GE 8 × 60 K Microarray
	GSE51618	Feng J (unpublished)	8 PitNET vs. 3 normal	GPL6480: Agilent-014850 Whole Human Genome Microarray 4 × 44 K G4112F
	GSE26966	Donsom AM [23]	14 PitNET vs. 9 normal	GPL570: Affymetrix Human Genome U133 Plus 2.0 Array
	GSE63357	Barry S [24,25,26]	16 PitNET vs. 5 normal	GPL570: Affymetrix Human Genome U133 Plus 2.0 Array
Pheochromocytoma	GSE50442	Shankavaram U (unpublished)	3 PHEO vs. 8 normal	GPL6244: Affymetrix Human Gene 1.0 ST Array
	GSE39716	Shankavaram U [27,28,29]	21 PHEO vs. 8 normal	GPL6244: Affymetrix Human Gene 1.0 ST Array
	GSE19422	López-Jiménez E [30]	61 PHEO vs. 6 normal	GPL6480: Agilent-014850 Whole Human Genome Microarray 4 × 44 K G4112F
	GSE60459	Choi Y-L (unpublished)	2 PHEO vs. 3 normal	GPL13607: Agilent-028004 SurePrint G3 Human GE 8x60K Microarray
Paraganglioma	GSE50442	Shankavaram U (unpublished)	4 PGL vs. 8 normal	GPL6244: Affymetrix Human Gene 1.0 ST Array
	GSE39716	Shankavaram U [27,28,29]	24 PGL vs. 8 normal	GPL6244: Affymetrix Human Gene 1.0 ST Array
	GSE19422	López-Jiménez E [30]	23 PGL vs. 6 normal	GPL6480: Agilent-014850 Whole Human Genome Microarray 4 × 44 K G4112F
	GSE60459	Choi Y-L (unpublished)	10 PGL vs. 3 normal	GPL13607: Agilent-028004 SurePrint G3 Human GE 8x60K Microarray
	GSE90713	Fraber JM (unpublished)	57 ACC vs. 4 normal	GPL15207: Affymetrix Human Gene Expression Array
	GSE14922	Szabó PM [31]	4 ACC vs. 4 normal	GPL6480: Agilent-014850 Whole Human Genome Microarray 4 × 44 K G4112F
	GSE12368	Soon PS [32]	12 ACC vs. 6 normal	GPL570: Affymetrix Human Genome U133 Plus 2.0 Array
	GSE19750	Bussey KJ [33,34]	44 ACC vs. 4 normal	GPL570: Affymetrix Human Genome U133 Plus 2.0 Array
	GSE33371	Heanton JH [35]	33 ACC vs. 10 normal	GPL570: Affymetrix Human Genome U133 Plus 2.0 Array

**Abbreviations**: Medullary thyroid cancer (MTC), Pheochromocytoma (PHEO), Paraganglioma (PGL), Pituitary neuroendocrine tumor (PitNET), Adrenocortical carcinoma (ACC).

**Table 2:** List of DEGs Selected from Analysis of Medullary Thyroid Cancer Datasets.

GPCR Gene Symbol	Log_2_FC	Adj-*p*-Val
*TSHR*	−2.22	0.0001
*GPRC5A*	−1.93	0.0002
*OPN3*	−0.98	0.0011
*FZD1*	−0.89	0.0005
*ADGRE5*	−0.84	0.0014
*F2RL1*	−0.67	0.0043
*FZD2*	−0.56	0.0041
*CCKBR*	0.59	0.0025
*DRD2*	0.62	0.0030
*ADORA2B*	0.64	0.0029
*CELSR3*	0.80	0.0032
*GPR19*	0.84	0.0021
*ADGRG2*	1.42	0.0049
*LGR5*	1.43	0.0003

**Table 3:** List of GPCRs with Approved Drugs.

GPCR	Mode of Action	Main Indication(s)
***ADORA2B***		
Caffeine	Antagonist	pulmonary complications of premature birth
Enprofylline	Antagonist	Asthma
Theophylline	Antagonist	Asthma
Adenosine	Agonist	supraventricular tachycardia
***AVPR1A***		
Atosiban	Antagonist	pre-term labour
Conivaptan	Antagonist	SIADH
Felypressin	Agonist	diabetes insipidus
Terlipressin	Agonist	bleeding caused by esophageal varices.
***CCKBR***		
Pentagastrin	Agonist	evaluation of gastric acid secretory function
***DRD2***		
>40 Drugs	Antagonist	antipsychotic agent
Alizapride	Antagonist	anti-emetic
Bromopride	Antagonist	anti-emetic
Domperidone	Antagonist	anti-emetic
Droperidol	Antagonist	anti-emetic
Metoclopramide	Antagonist	anti-emetic
8 Drugs *	Agonist	Parkinson’s disease
Cabergoline	Agonist	pituitary adenoma
Quinagolide	Agonist	pituitary adenoma
***HTR4***		
Vilazodone	Agonist	Depressive disorder
Mosapride	Agonist	Gastroprokinetic agent
Cisapride	Agonist	Gastroprokinetic agent
Prucalopride	Agonist	Gastroprokinetic agent
Cinitapride	Agonist	Gastroprokinetic agent
Tegaserod	Agonist	Gastroprokinetic agent
Metoclopramide	Antagonist	anti-emetic
***MC2R***		
Corticotropin	Agonist	diagnosis agent
Tetracosactide	Agonist	diagnosis agent
***PTGER2***		
Alprostadil	Agonist	erectile dysfunction
Dinoprostone	Agonist	labor induction
Misoprostol	Agonist	gastric ulcer
Gemeprost	Agonist	pregnancy termination
Limaprost	Agonist	Ischemic ulcer
***PTH1R***		
Abaloparatide	Agonist	Osteoporosis
Parathyroid hormone	Agonist	Hypoparathyroidism
Teriparatide	Agonist	Osteoporosis
***SSTR1***		
Octreotide	Agonist	NeuroEndocrine tumor
Pasireotide	Agonist	NeuroEndocrine tumors

For each endocrine tumor, common dysregulated GPCR were reviewed and approved drugs were listed with their mode of action and their main indications. Abbreviations: SIADH = Syndrome of inappropriate antidiuretic hormone secretion. *: The eight DRD2 agonist were: Amantadine, apomorphine, Levodopa, lisuride, pergolide, pramipexole, ropinirole, rotigotine.

## Data Availability

The datasets generated and/or analyzed during the current study are available from the corresponding author on reasonable request.

## Supplementary Materials

The following are available online at https://www.mdpi.com/article/10.3390/cells11040703/s1, Table S1: List of GPCR genes included in bioinformatics analysis. Table S2: List of DEGs selected from analysis of pituitary adenoma datasets. Table S3: List of DEGs selected from analysis of pheochromocytoma datasets. Table S4. List of DEGs selected from analysis of paraganglioma datasets. Table S5: List of DEGs selected from analysis of adrenocortical cancer datasets.
