# Supplementary material for: Identification of Dysregulated Expression of G Protein Coupled Receptors in Endocrine Tumors by Bioinformatics Analysis: Potential Drug Targets?
Source: Cells. 2022 Feb 17;11(4):703. doi: 10.3390/cells11040703 (PMC8870215; doi:10.3390/cells11040703)
Supplement: Supplementary file 1 [file cells-11-00703-s001.zip › cells-1577003-supplementary.pdf]

Supplemental Table S1. List of GPCR genes included in bioinformatics analysis.

ACKR1	*ADGRG7*	*BRS3*	*CHRM5*	*FFAR2*
ACKR2	*ADGRL1*	*C3AR1*	*CMKLR1*	*FFAR3*
ACKR3	*ADGRL2*	*C5AR1*	*CNR1*	*FFAR4*
ACKR4	*ADGRL3*	*C5AR2*	*CNR2*	*FPR1*
ADCYAP1R1	*ADGRL4*	*CALCR*	*CRHR1*	*FPR2*
ADGRA1	*ADGRV1*	*CALCRL*	*CRHR2*	*FPR3*
ADGRA2	*ADORA1*	*CASR*	*CX3CR1*	*FSHR*
ADGRA3	*ADORA2A*	*CCKAR*	*CXCR1*	*FZD1*
ADGRB1	*ADORA2B*	*CCKBR*	*CXCR2*	*FZD10*
ADGRB2	*ADORA3*	*CCR1*	*CXCR3*	*FZD2*
ADGRB3	*ADRA1A*	*CCR10*	*CXCR4*	*FZD3*
ADGRD1	*ADRA1B*	*CCR2*	*CXCR5*	*FZD4*
ADGRE1	*ADRA1D*	*CCR3*	*CXCR6*	*FZD5*
ADGRE2	*ADRA2A*	*CCR4*	*CYSLTR1*	*FZD6*
ADGRE3	*ADRA2B*	*CCR5*	*CYSLTR2*	*FZD7*
ADGRE5	*ADRA2C*	*CCR6*	*DRD1*	*FZD8*
ADGRF1	*ADRB1*	*CCR7*	*DRD2*	*FZD9*
ADGRF2	*ADRB2*	*CCR8*	*DRD3*	*GABBR1*
ADGRF3	*ADRB3*	*CCR9*	*DRD4*	*GABBR2*
ADGRF4	*AGTR1*	*CCRL2*	*DRD5*	*GALR1*
ADGRF5	*AGTR2*	*CELSR1*	*EDNRA*	*GALR2*
ADGRG1	*APLNR*	*CELSR2*	*EDNRB*	*GALR3*
ADGRG2	*AVPR1A*	*CELSR3*	*F2R*	*GCGR*
ADGRG3	*AVPR1B*	*CHRM1*	*F2RL1*	*GHRHR*
ADGRG4	*AVPR2*	*CHRM2*	*F2RL2*	*GHSR*
ADGRG5	*BDKRB1*	*CHRM3*	*F2RL3*	*GIPR*
ADGRG6	*BDKRB2*	*CHRM4*	*FFAR1*	*GLP1R*

GLP2R	*GPR160*	*GPR4*	*GRM6*	*LHCGR*
GNRHR	*GPR161*	*GPR45*	*GRM7*	*LPAR1*
GNRHR2	*GPR162*	*GPR50*	*GRM8*	*LPAR2*
GPBAR1	*GPR17*	*GPR52*	*GRPR*	*LPAR3*
GPER1	*GPR171*	*GPR55*	*HCAR1*	*LPAR4*
GPR1	*GPR173*	*GPR6*	*HCAR2*	*LPAR5*
GPR1	*GPR174*	*GPR61*	*HCAR3*	*LPAR6*
GPR101	*GPR176*	*GPR62*	*HCRTR1*	*LTB4R*
GPR107	*GPR179*	*GPR63*	*HCRTR2*	*LTB4R2*
GPR119	*GPR18*	*GPR65*	*HRH1*	*MAS1*
GPR12	*GPR182*	*GPR68*	*HRH2*	*MAS1L*
GPR132	*GPR183*	*GPR75*	*HRH3*	*MC1R*
GPR135	*GPR19*	*GPR78*	*HRH4*	*MC2R*
GPR137	*GPR20*	*GPR82*	*HTR1A*	*MC3R*
GPR139	*GPR20*	*GPR83*	*HTR1B*	*MC4R*
GPR141	*GPR21*	*GPR84*	*HTR1D*	*MC5R*
GPR142	*GPR22*	*GPR85*	*HTR1E*	*MCHR1*
GPR143	*GPR25*	*GPR87*	*HTR1F*	*MCHR2*
GPR146	*GPR26*	*GPR88*	*HTR2A*	*MLNR*
GPR148	*GPR27*	*GPRC5A*	*HTR2B*	*MRGPRD*
GPR149	*GPR3*	*GPRC5B*	*HTR2C*	*MRGPRE*
GPR15	*GPR31*	*GPRC5C*	*HTR4*	*MRGPRF*
GPR150	*GPR32*	*GPRC5D*	*HTR5A*	*MRGPRG*
GPR151	*GPR33*	*GPRC6A*	*HTR6*	*MRGPRX1*
GPR152	*GPR34*	*GRM1*	*HTR7*	*MRGPRX2*
GPR153	*GPR35*	*GRM2*	*KISS1R*	*MRGPRX3*
GPR156	*GPR37*	*GRM3*	*LGR4*	*MRGPRX4*
GPR157	*GPR37L1*	*GRM4*	*LGR5*	*MTNR1A*
GPR158	*GPR39*	*GRM5*	*LGR6*	*MTNR1B*

NMBR	*OXER1*	*PTGIR*	*TAAR3*	*TAS2R39*
NMUR1	*OXGR1*	*PTH1R*	*TAAR5*	*TAS2R4*
NMUR2	*OXTR*	*PTH2R*	*TAAR6*	*TAS2R40*
NPBWR1	*P2RY1*	*QRFPR*	*TAAR8*	*TAS2R41*
NPBWR2	*P2RY10*	*RXFP1*	*TAAR9*	*TAS2R42*
NPBWR2	*P2RY11*	*RXFP2*	*TACR1*	*TAS2R43*
NPFFR1	*P2RY12*	*RXFP3*	*TACR2*	*TAS2R46*
NPFFR2	*P2RY13*	*RXFP4*	*TACR3*	*TAS2R5*
NPSR1	*P2RY14*	*S1PR1*	*TAS1R1*	*TAS2R50*
NPY1R	*P2RY2*	*S1PR2*	*TAS1R2*	*TAS2R60*
NPY2R	*P2RY4*	*S1PR3*	*TAS1R3*	*TAS2R7*
NPY4R	*P2RY6*	*S1PR4*	*TAS2R1*	*TAS2R8*
NPY5R	*P2RY8*	*S1PR5*	*TAS2R10*	*TAS2R9*
NPY6R	*PRLHR*	*SCTR*	*TAS2R13*	*TBXA2R*
NTSR1	*PROKR1*	*SMO*	*TAS2R14*	*TPRA1*
NTSR2	*PROKR2*	*SSTR1*	*TAS2R16*	*TRHR*
OPN3	*PTAFR*	*SSTR2*	*TAS2R19*	*TSHR*
OPN4	*PTGDR*	*SSTR3*	*TAS2R19*	*UTS2R*
OPN5	*PTGDR2*	*SSTR4*	*TAS2R20*	*VIPR1*
OPRD1	*PTGER1*	*SSTR5*	*TAS2R20*	*VIPR2*
OPRK1	*PTGER2*	*SUCNR1*	*TAS2R3*	*XCR1*
OPRL1	*PTGER3*	*TAAR1*	*TAS2R30*	
OPRM1	*PTGER4*	*TAAR2*	*TAS2R31*	
OR51E1	*PTGFR*		*TAS2R38*	

Supplemental Table S2. List of DEG selected from analysis of Pituitary adenoma datasets

**GSE119063**	**GSE51618**	**GSE36314**	**GSE63357**	**GSE26966**
ADRA1B	*ADGRG5*	*FZD7*	*ADGRB3*	*ACKR1*
AVPR1B	*ADGRL1*		*CX3CR1*	*ADGRB2*
CCKBR	*CCR6*		*DRD2*	*ADGRD1*
CCR5	*CRHR1*		*FZD1*	*ADGRE5*
CRHR1	*CRHR2*		*FZD7*	*ADGRG2*
CRHR1	*DRD3*		*FZD9*	*ADGRL1*
CRHR2	*FZD7*		*GALR1*	*ADGRL3*
FFAR4	*GALR3*		*GNRHR*	*ADGRL4*
FSHR	*GHRHR*		*GPR153*	*ADORA3*
FZD1	*GIPR*		*GPR161*	*ADRA2A*
FZD5	*GNRHR*		*GPR19*	*APLNR*
FZD7	*GNRHR2*		*GPR26*	*CRHR1*
FZD9	*GPR135*		*GPR3*	*CX3CR1*
GALR1	*GPR137*		*GPR34*	*CYSLTR1*
GHRHR	*GPR176*		*GRM5*	*DRD2*
GIPR	*GPR45*		*LGR6*	*F2R*
GNRHR	*GPR62*		*MC1R*	*F2RL1*
GPR12	*HRH2*			*FFAR4*
GPR149	*HTR1F*			*FPR1*
GPR152	*HTR4*			*FZD1*
GPR45	*LGR6*			*FZD10*
GPR54	*MC2R*			*FZD3*
GPR6	*MRGPRX4*			*FZD7*

GSE119063	GSE51618	GSE36314	GSE63357	GSE26966
GPR62	*NPBWR1*			*GABBR1*
HTR2B	*NPFFR2*			*GALR1*
HTR5A	*NTSR1*			*GHRHR*
LGR4	*P2RY1*			*GHSR*
LPAR6	*SSTR2*			*GNRHR*
MC2R	*SSTR3*			*GPR135*
NPY1R	*SSTR5*			*GPR143*
NPY2R	*TAAR8*			*GPR153*
NPY5R	*VN1R5*			*GPR158*
OXER1				*GPR160*
P2RY1				*GPR161*
P2RY12				*GPR173*
PTGER1				*GPR176*
SSTR2				*GPR22*
SSTR5				*GPR27*
VIPR2				*GPR34*
				GPR6
				GPR61
				GPR68
				GPR75
				GPR85
				GPRC5C
				GRM1
				GRM5

GSE119063	GSE51618	GSE36314	GSE63357	GSE26966
				GRM8
				HRH3
				HTR6
				LGR6
				LPAR6
				MATK
				MC1R
				NPY6R
				OPN3
				P2RY1
				P2RY12
				P2RY13
				PRLHR
				PTGER3
				PTGER4
				S1PR1
				S1PR3
				SSTR2
				TBXA2R

Supplemental Table S3. List of DEG selected from analysis of pheochromocytoma datasets

GSE50442	GSE39716	GSE19422	GSE60459
GRM7	*GRM7*	*GPR158*	*ADGRD1*
HRH1	*NPY5R*	*GPR19*	*S1PR5*
GPR137C	*EDNRA*	*P2RY2*	*GPR18*
GPR4	*LHCGR*	*ADGRB3*	*CCR8*
CNR1	*GPR4*	*MC2R*	*HTR2B*
P2RY8	*HCRTR1*	*ADGRF4*	*GPR143*
ADRB2	*ADGRF4*	*ADGRG5*	*NPY1R*
CX3CR1	*CHRM1*	*OR7E5P*	*GPR82*
LHCGR	*CX3CR1*	*TBXA2R*	*FZD1*
EDNRA	*CNR1*	*GALR1*	*FSHR*
GHSR	*HRH1*	*LGR4*	*CASR*
ADGRF4	*P2RY12*	*NPY6R*	*GRM3*
	HTR2B	*GPR68*	*GPR52*
	LGR4	*OPN4*	*VIPR2*
	S1PR1	*PTGER3*	*MC2R*
	GHSR	*CELSR3*	*CMKLR1*
	AGTR2	*ADGRL3*	*GPR182*
	APLNR	*ADGRV1*	*P2RY12*
	ACKR3	*ADGRA1*	*RHO*
	F2RL3	*GPR176*	*CXCR3*
	ADGRG7	*NTSR2*	*MTNR1B*
	SSTR2	*SMO*	*F2RL2*
	GABBR2	*GNRHR*	*TRHR*
	CALCR	*GRM3*	*AVPR1B*

GSE50442	GSE39716	GSE19422	GSE60459
	CALCRL	*HCRTR1*	*HTR4*
	CHRM3	*AVPR1A*	*AGTR2*
	BDKRB2	*GRM8*	*CCR5*
	FZD5	*GPR20*	*NPY2R*
	GPR20	*GPR22*	*GNRHR*
	RXFP1	*CCR10*	*FPR3*
	GPR21	*FZD4*	*SMO*
	ADGRA2	*AGTR2*	*ACKR4*
	ADGRL4	*GPR135*	*FFAR1*
	CMKLR1	*GPR137C*	*GPR3*
	HTR1F	*AGTR1*	*TACR3*
	MCHR1	*SSTR1*	
	ACKR2	*ADGRE5*	
	P2RY8	*CHRM3*	
	ADGRL3	*OPRK1*	
	C3AR1	*FZD9*	
	GPR65	*PTH1R*	
	GPR52	*RRH*	
	S1PR5	*GABBR1*	
	GPR3	*NPY5R*	
	GPR82	*FZD3*	
	GPR17	*GPR27*	
	GPR63	*GRIPAP1*	
		ADGRL1	

GSE50442	GSE39716	GSE19422	GSE60459
		CHRM2	
		GPR25	
		P2RY8	
		LPAR1	
		S1PR3	
		MCHR2	
		HPRT1	
		PRLHR	
		ADGRF1	
		OPN3	
		PTGER2	
		GPR143	
		HTR1E	
		GPR61	
		GPR65	
		F2RL3	
		ACKR3	
		MCHR1	
		LTB4R2	
		CCR1	
		GPR83	
		MC1R	
		ADORA3	
		NMUR1	

GSE50442	GSE39716	GSE19422	GSE60459
		CCR6	
		CHRM5	
		RXFP1	
		GPRC5A	
		FZD2	
		FPR2	
		DRD2	
		PTGER4	
		OPRM1	
		SUCNR1	
		CCR3	
		CCR4	
		HRH1	
		GPR119	

Supplemental Table S4. List of DEG selected from analysis of paraganglioma datasets

**GSE50442**	**GSE39716**	**GSE19422**	**GSE60459**
ACKR3	*ACKR3*	*ACKR3*	*ACKR4*
ADCYAP1R1	*ADGRA1*	*ADGRA2*	*ADGRA3*
ADGRE5	*ADGRA2*	*ADGRB3*	*ADGRG4*
ADGRG6	*ADGRB3*	*ADGRE5*	*ADGRV1*
ADGRL3	*ADGRF4*	*ADGRF4*	*AGTR2*
ADGRV1	*ADGRG7*	*ADGRG5*	*AVPR1A*
AVPR1A	*ADGRL1*	*ADGRL1*	*AVPR1B*
C3AR1	*ADGRL4*	*ADGRL3*	*CASR*
CALCRL	*AGTR1*	*ADGRV1*	*CCKBR*
CMKLR1	*AGTR2*	*ADORA2A*	*CCR5*
CNR1	*APLNR*	*ADORA3*	*CCR8*
CX3CR1	*AVPR1A*	*AGTR1*	*CCR9*
CYSLTR2	*BDKRB2*	*AVPR1A*	*CHRM5*
F2RL2	*C3AR1*	*BDKRB1*	*CMKLR1*
FZD5	*CHRM1*	*BDKRB2*	*CNR2*
GPR1	*CHRM3*	*CALCRL*	*CXCR3*
GPR137B	*CX3CR1*	*CELSR3*	*DRD1*
GPR19	*EDNRA*	*CHRM2*	*FFAR1*
GRM7	*F2R*	*CHRM3*	*FSHR*
HRH1	*F2RL1*	*EDNRA*	*GHRHR*
HTR1E	*F2RL3*	*F2RL3*	*GNRHR*
HTR2B	*FZD10*	*FZD3*	*GPR158*
HTR4	*FZD5*	*FZD9*	*GPR182*
LGR4	*FZD6*	*GABBR1*	*GPR182*

**GSE50442**	**GSE39716**	**GSE19422**	**GSE60459**
MC2R	*GALR1*	*GNRHR*	*GPR19*
MCHR1	*GHSR*	*GPER1*	*GPR45*
NPY5R	*GPR137*	*GPR12*	*GPR52*
NPY6R	*GPR143*	*GPR135*	*GPR63*
OPN3	*GPR158*	*GPR137C*	*GPR82*
OPRK1	*GPR161*	*GPR158*	*GRM3*
OPRM1	*GPR173*	*GPR176*	*GRM5*
OPRM1	*GPR20*	*GPR19*	*GRM7*
OXTR	*GPR21*	*GPR20*	*HRH4*
P2RY12	*GPR34*	*GPR22*	*HTR2B*
PTGER2	*GPR4*	*GPR25*	*HTR2C*
PTH1R	*GPR52*	*GPR27*	*LGR4*
RXFP1	*GPR63*	*GPR61*	*LHCGR*
SSTR2	*GPR82*	*GPR78*	*MC1R*
	GPRC5B	*GPR83*	*MC2R*
	GPRC5C	*GRM8*	*MCHR1*
	GRM7	*HCRTR1*	*MLNR*
	HCRTR1	*HTR1E*	*MTNR1A*
	HRH1	*KISS1R*	*MTNR1B*
	HTR2B	*LGR4*	*NPFFR1*
	LGR4	*LPAR1*	*NPY1R*
	LGR5	*LPAR3*	*NPY2R*
	LHCGR	*LTB4R2*	*OPN3*
	MC2R	*MC1R*	*P2RY1*

GSE50442	GSE39716	GSE19422	GSE60459
	NPBWR1	*MC2R*	*P2RY11*
	NPY5R	*MCHR1*	*PTH2R*
	NPY6R	*NPY1R*	*PTHR1*
	OPRK1	*NPY5R*	*RHO*
	OPRM1	*NPY6R*	*RXFP2*
	PTGER2	*NTSR2*	*RXFP3*
	PTH1R	*OPN4*	*S1PR3*
	RXFP1	*OPRK1*	*S1PR5*
	S1PR1	*OR7E5P*	*SSTR3*
		P2RY14	*SUCNR1*
		P2RY2	*TACR2*
		PRLHR	*TACR3*
		PTGER2	*TBXA2R*
		PTGER3	*VN1R1*
		PTH1R	*VN1R2*
		RRH	*NPY5R*
		RXFP1	*NPY6R*
		SMO	*RXFP1*
		SSTR2	
		SSTR5	
		TBXA2R	

Supplemental Table S5. List of DEG selected from analysis of adrenocortical cancer datasets

**GSE14922**	**GSE12368**	**GSE19750**	**GSE33371**	**GSE90713**
ACKR4	*ADGRB3*	*ACKR1*	*ACKR1*	*ADGRE2*
CMKLR1	*ADORA3*	*ADCYAP1R1*	*ACKR2*	*ADORA3*
CXCR5	*CCR10*	*ADGRF5*	*ADGRA1*	*CMKLR1*
HTR2B	*FFAR4*	*ADRB1*	*ADGRF5*	*FZD1*
PTH1R	*GRM3*	*CXCR2*	*ADGRG6*	*GPR182*
RXFP2	*MC1R*	*DRD2*	*ADGRL1*	*HTR2B*
	PRLHR	*GPR182*	*ADORA3*	*MC2R*
	PTGER4	*GPR68*	*ADRA1A*	*PTH1R*
	PTH1R	*GPR84*	*AGTR2*	
		HTR4	*C3AR1*	
		MC2R	*C5AR1*	
		PTGER4	*CCR1*	
		PTGIR	*CCR8*	
		PTH1R	*CMKLR1*	
		SSTR2	*FPR1*	
			FPR3	
			FZD1	
			FZD2	
			FZD9	
			GABBR1	
			GLP1R	
			GPBAR1	
			GPR135	
			GPR137C	
			GPR160	
			GPR182	

GSE14922	GSE12368	GSE19750	GSE33371	GSE90713
			GPR34	
			GPR65	
			GPR82	
			GPR84	
			GRM3	
			HTR1E	
			HTR2B	
			LGR4	
			LPAR6	
			LTB4R	
			MC1R	
			MC2R	
			MCHR1	
			OPN5	
			OXTR	
			P2RY12	
			P2RY13	
			P2RY14	
			PTAFR	
			PTGER3	
			PTGER4	
			PTGIR	
			PTH1R	
			PTPN22	
			S1PR1	
			VN1R1	
